# Supplementary figures and images for: Characterization of the salivary microbiome in patients with pancreatic cancer
Source: PeerJ. 2015 Nov 5;3:e1373. doi: 10.7717/peerj.1373 (PMC4647550; doi:10.7717/peerj.1373)

a.

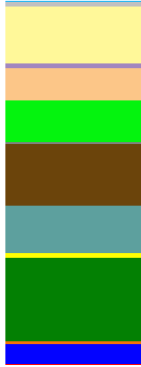

b.

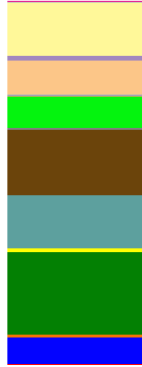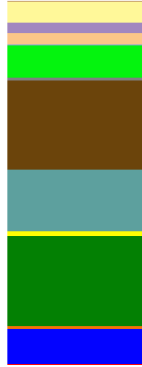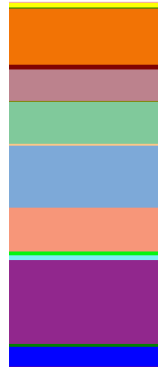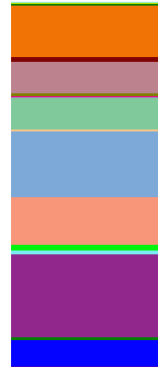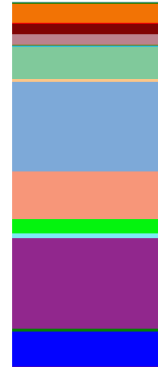

c.

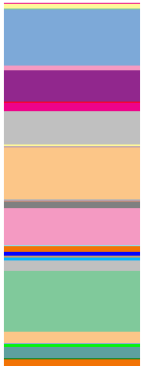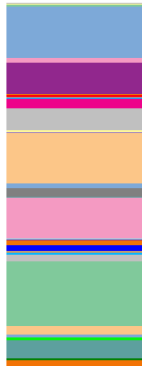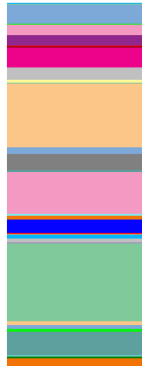

d.

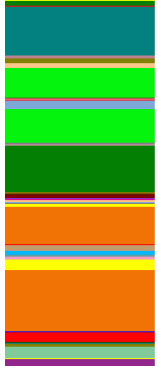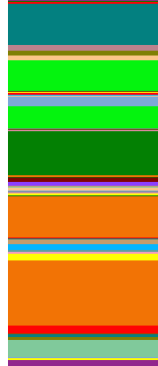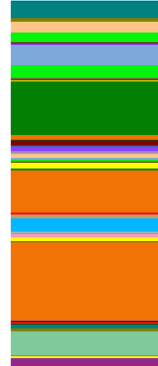

Supplement: Figure S1 — Relative abundance of (A) class, (B) order, (C) family, (D) genus of the oral communities from 108 study patients according to diagnosis. Patients are grouped by diagnosis (H, healthy; O, other; P, pancreatic cancer). [file peerj-03-1373-s001.pdf]
